# Fundus features of acute macular neuroretinopathy after SARSCoV2 infection: A multimodal imaging analysis

**DOI:** 10.1097/MD.0000000000049217

**Published:** 2026-09-25

**Authors:** Chao Sun, Huaihua Wang, Na Liu, Hui Lu, Shengnan Zhang

**Affiliations:** a Department of Ophthalmology, Zibo Central Hospital, Zibo City, Shandong Province, China.

**Keywords:** Acute macular neuroretinopathy, COVID19, infrared reflectance imaging, mfERG, optical coherence tomography, optical coherence tomography angiography

## Abstract

Acute macular neuroretinopathy (AMN) is a rare macular disease. We aimed to report the fundus multimodal imaging features of AMN with SARSCoV2 infection. Six consecutive cases of AMN occurred in our hospital from December 2022 to January 2023, all within one week after COVID-19 infection. 6 patients (12 eyes) diagnosed as AMN with SARSCoV2 infection were selected. All 6 patients developed high fever post-SARS-CoV-2 infection, with sudden central/paracentral scotoma appearing 2–4 days later as the main visual symptom. Fundus examination showed normal fundus in 4 eyes and macular foveal reflection loss in 8 eyes. The infrared reflectance imaging demonstrated localized areas of hyporeflection in or around the macula, among them, 7 eyes had hyporeflection lesions located in fovea of macula, and 5 eyes were located on the nasal side of fovea of macula. The fluorescein fundus angiography (FFA) revealed no vascular pathology. In all eyes, optical coherence tomography (OCT) shows hyperreflective in the photoreceptor layers, the external limiting membrane, outer nuclear layer up to the outer plexiform layer, and defect involving the ellipsoid and interdigitation zone. Optical coherence tomography angiography (OCTA) shows deep capillary plexus (DCP) vascular texture was diffusely sparse in 1 eye; enface imaging shows high reflection in the corresponding area. No difference was observed in imaging features between AMN after SARS-CoV-2 infection and AMN not related to SARS-CoV-2. However, the increasing cases after COVID-19 infection imply that patients with SARS-CoV-2 infection are at greater risk of developing AMN.

## 1. Introduction

Acute macular neuroretinopathy (AMN) is a rare macular disease first described by Bos et al^[1]^ in 1975, affecting the outer retina of the macular region. Patients mainly present with dark spots in the center or paracentral region of one or both eyes and may experience mild vision loss. In fundus examination, we can see circular, quasi-circular, wedge-shaped, or petal-like lesions near the macular center. The causes of AMN are diverse, including oral contraceptives, virus infection, epinephrine application, head trauma, shock, hypotension, COVID-19 vaccine injection, and leukemia chemotherapy.^[2,3]^

As the COVID-19 control policy lifted in December 2022, Omicron infection cases soared from December 2022 to January 2023 in China. At the same time, we noticed that the number of patients diagnosed with AMN increased obviously (in the past decade, there were only 0–2 new cases per year), implying the correlation between AMN and COVID-19 infection. Due to the low incidence rate of AMN, the understanding of the image changes of AMN is limited, which often leads to misdiagnosis. Since the incidence rate of AMN has increased in a short time after SARS-CoV-2 infection, we have a chance to better observe the imaging changes related to AMN. Therefore, we reported 6 cases of patients diagnosed with AMN after SARS-CoV-2 infection in our hospital between December 2022 and January 2023. We conducted an in-depth analysis of the multimodal imaging of the fundus of these patients, hoping to better understand the fundus features of AMN with SARS-CoV-2 infection.

## 2. Materials and methods

### 2.1. Patient information

This study was approved by the Ethics Committee of Zibo Central Hospital.This is a retrospective study, selecting 6 cases (12 eyes) of AMN patients with SARS-CoV-2 infection in our hospital from December 2022 to February 2023 as the research subjects. Among them, 2 were male patients and 4 were female patients; the patients’ age at onset ranged from 21 to 49 years old, with an average age of (33.0 ± 10.0) years old.

All selected patients met the AMN diagnostic criteria proposed by Fawzi et al,^[4]^ including: a history of acute central or paracentral scotoma, possibly accompanied by vision loss; a normal result in fluorescence fundus angiography (FFA); near-infrared fundus photography showing a weak reflective lesion in the macular region; optical coherence tomography (OCT) showing focal point-like high reflection in the outer nuclear layer, consistent with the weak reflective lesion range in the macular region shown by near-infrared fundus photography. All patients were positive for SARS-CoV-2 antigen testing.

This study strictly followed the ethical principles required by the Helsinki Declaration, and all patients signed informed consent forms and passed the ethical review of our hospital ethics committee.

### 2.2. Clinical examination

All patients underwent best corrected visual acuity (BCVA), intraocular pressure, slit-lamp examination, and ultra-widefield fundus imaging. After sufficient pupil dilation with compound tropicamide eye drops, indirect ophthalmoscopy or a 90D anterior condensing lens was used for fundus examination. In addition, a Japanese KOWA fundus camera was used to take fundus color photos, a German Heidelberg HRA2 diagnostic device was used for near-infrared fundus photography, FFA (all patients underwent skin testing before angiography), and OCT examination, and an American Optovue optical coherence tomography scanner was used for OCTA examination. Based on the results of fundus color photos, near-infrared fundus photography, FFA, OCT, and OCTA examination of the patients, we analyzed the clinical characteristics of SARS-CoV-2 infection-related AMN.

## 3. Results

### 3.1. Clinical characteristics

All patients received 3 doses of inactivated COVID-19 vaccine before infection with SARS-CoV-2. All 6 patients presented with high fever and cough following SARS-CoV-2 infection. Within 2 to 4 days (2.8 ± 0.7 days) after the fever appeared, they developed dark spots in the center or paracentral area of one or both eyes, accompanied by vision loss. Upon admission, the patients’ BCVA was 0.60 ± 0.24, and their intraocular pressure was within the normal range. No significant abnormalities were observed in the anterior segment of both eyes, as shown in Table 1.

**Table 1:** Clinical characteristics.

Case No. (first diagnosis time)	Sex	Age (y)	SARS-CoV2 related symptoms	logMAR	Onset of ocular symptom (d)	follow-up (d)
				BCVA		
1	Female	49	fever, cough	1	3	19
				1		
2	Female	21	fever, cough	0.2	4	130
				1		
3	Male	38	fever, cough	0.7	3	103
				0.8		
4	Female	29	fever, cough	0.7	2	206
				0.6		
5	Female	39	fever, cough	0.4	3	135
				0.6		
6	Female	22	fever, cough	0.6	2	30

### 3.2. Typical manifestations of multimodal images

The results of fundus examination showed that the fundus of 4 eyes was normal, while the fundus of 8 eyes presented with disappearance of light reflex and local darkness in the macular region. The results of near-infrared fundus examination showed that all affected eyes presented with focal, weak reflective lesions in the perifoveal area, with 7 eyes having lesions located at the foveal center and 5 eyes having lesions located at the nasal side of the foveal center. In case 1, case 4, and case 6, both eyes presented with disappearance of light reflex and local darkness in the macular area. In case 5, the lesion of the left eye was located at the nasal side of the foveal center, and the lesion of the right eye was located at the macular area. Near-infrared fundus photography showed irregular low-reflective lesions around the foveal center in case 1, case 4, and case 6. Figure 1 showed the images of fundus examination and near-infrared fundus photography in cases 1, 4, 5 and 6.

**Figure 1.**
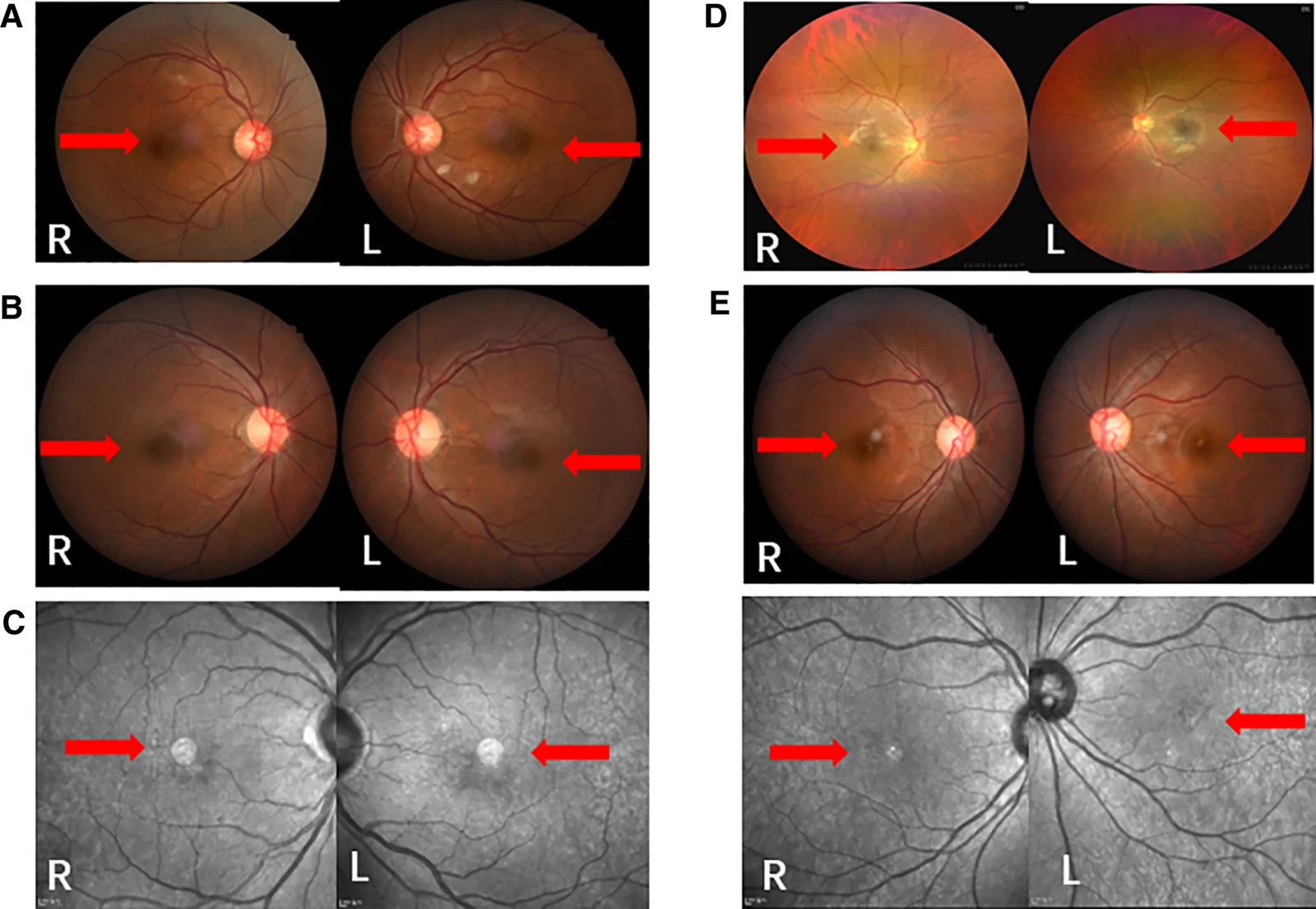
Fundus color photography and near-infrared fundus photography. (A)Fundus color photography of case 1. (B) Fundus color photography of case 4. (C) Near-infrared fundus photography of case 4. (D) Fundus color photography of case 5. (E) Fundus color photography of case 6. (F) Near-infrared fundus photography of case 6. R, right eye. L, left eye. Red arrow, the area with disappearance of light reflex and local darkness.

Ultra-wide field fundus imaging was performed in case 2 and case 4. In the wide-angle fundus images of these 2 cases, apart from the appearance of AMN, no other SARS-CoV-2 infection-related fundus manifestations were observed (Fig. 2).

**Figure 2.**
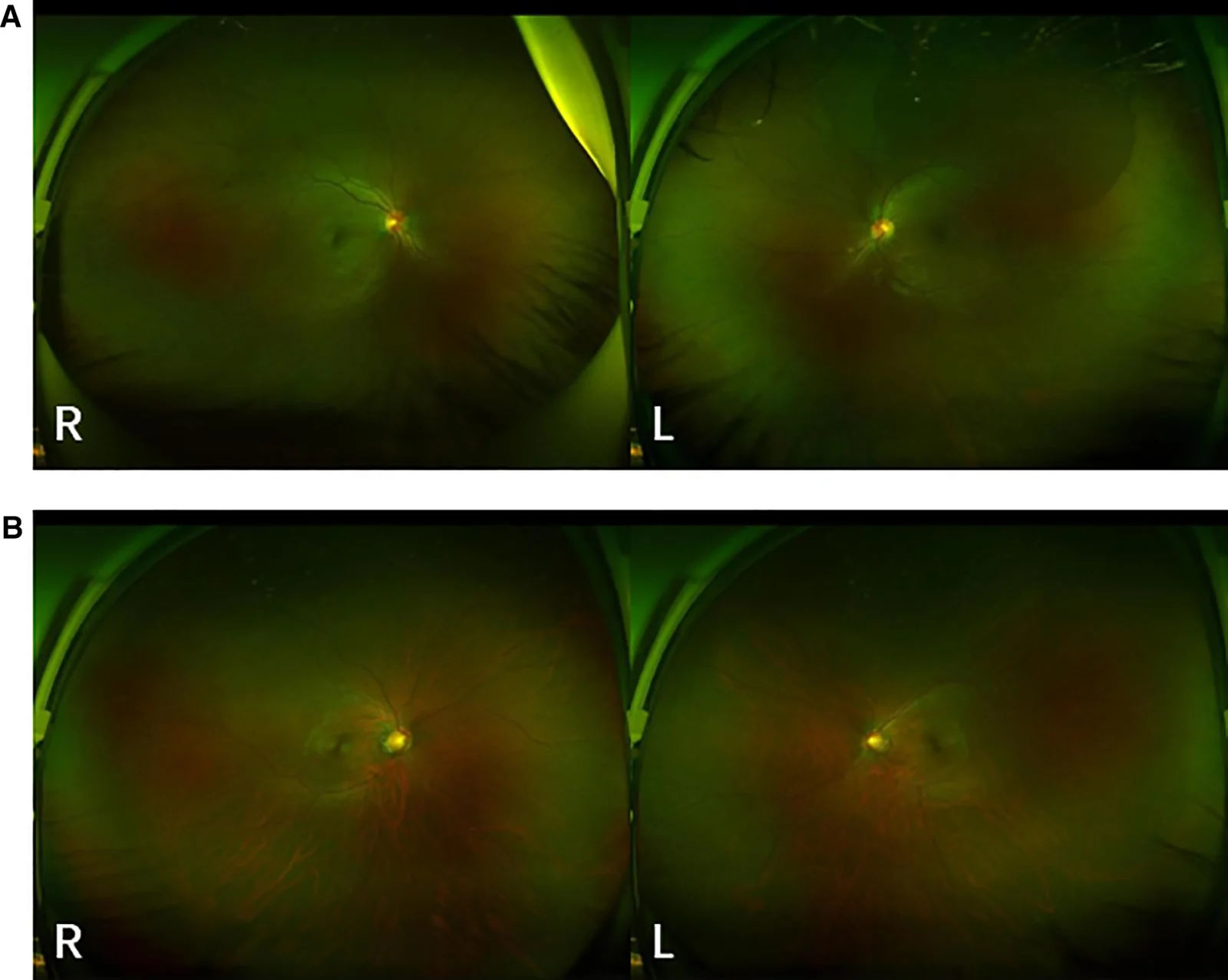
Ultra-wide field fundus images of case 2 and case 4. (A) Case 2. (B) Case 4. L = left eye, R = right eye.

The OCT examination results showed that all affected eyes presented with focal, high-reflective lesions in the outer nuclear layer and outer plexiform layer, as well as local damage to the ellipsoid zone and interdigitation zone (Fig. 3).

**Figure 3.**
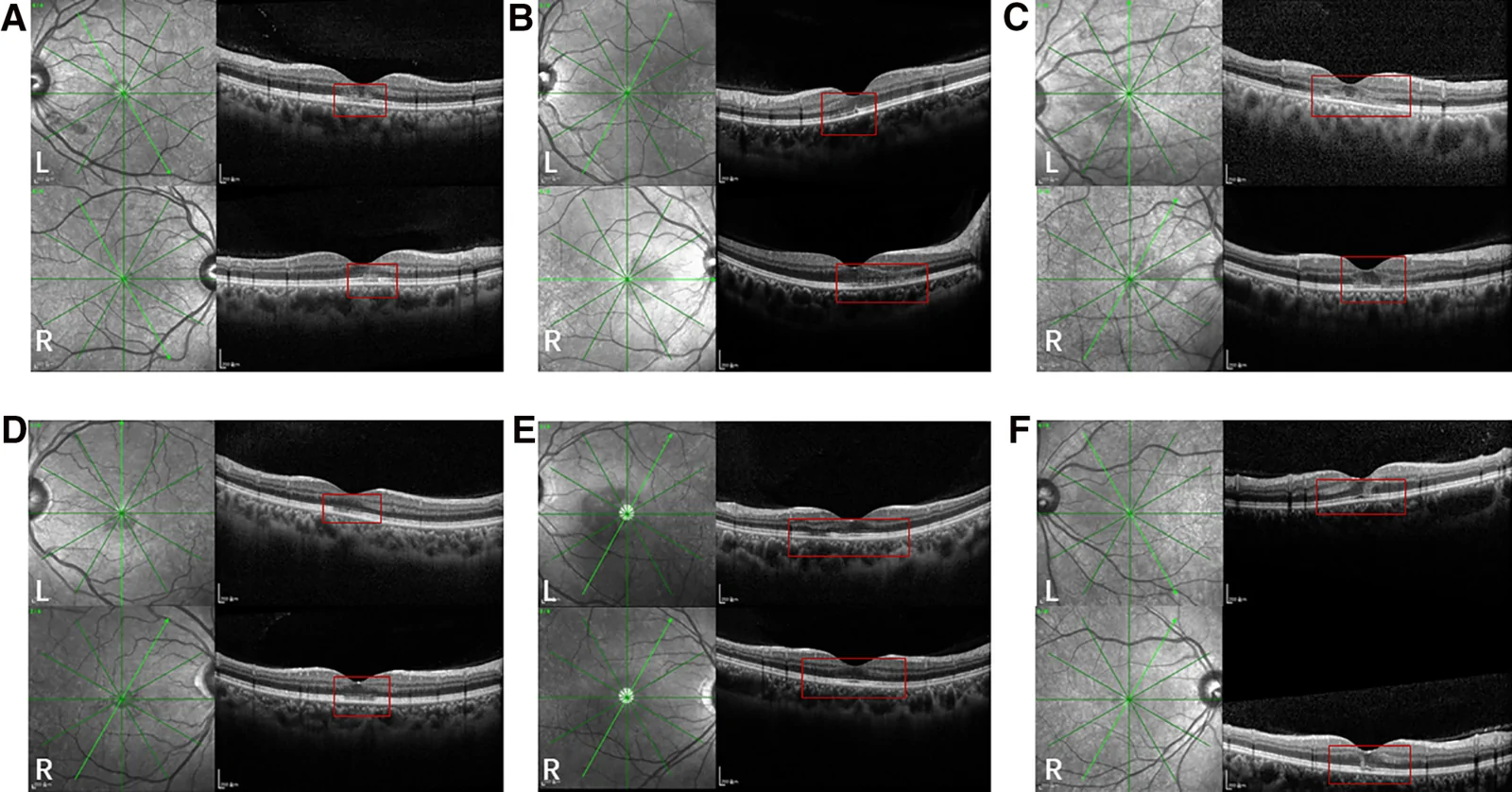
OCT B-scan image of Cases 1 to 6. High reflectivity in the outer plexiform layer and outer nuclear layer in the macular region, with defects in the ellipsoid zone and IZ band (red rectangle). (A) Case 1. (B) Case 2. (C) Case 3. (D) Case 4. (E) Case 5. (F) Case 6. L = left eye, R = right eye.

The OCTA examination results revealed that one eye presented with a reduced blood flow density in the deep retinal capillaries, and enface OCT scanning at the corresponding location revealed a high reflection in case 1 (Fig. 4).

**Figure 4.**
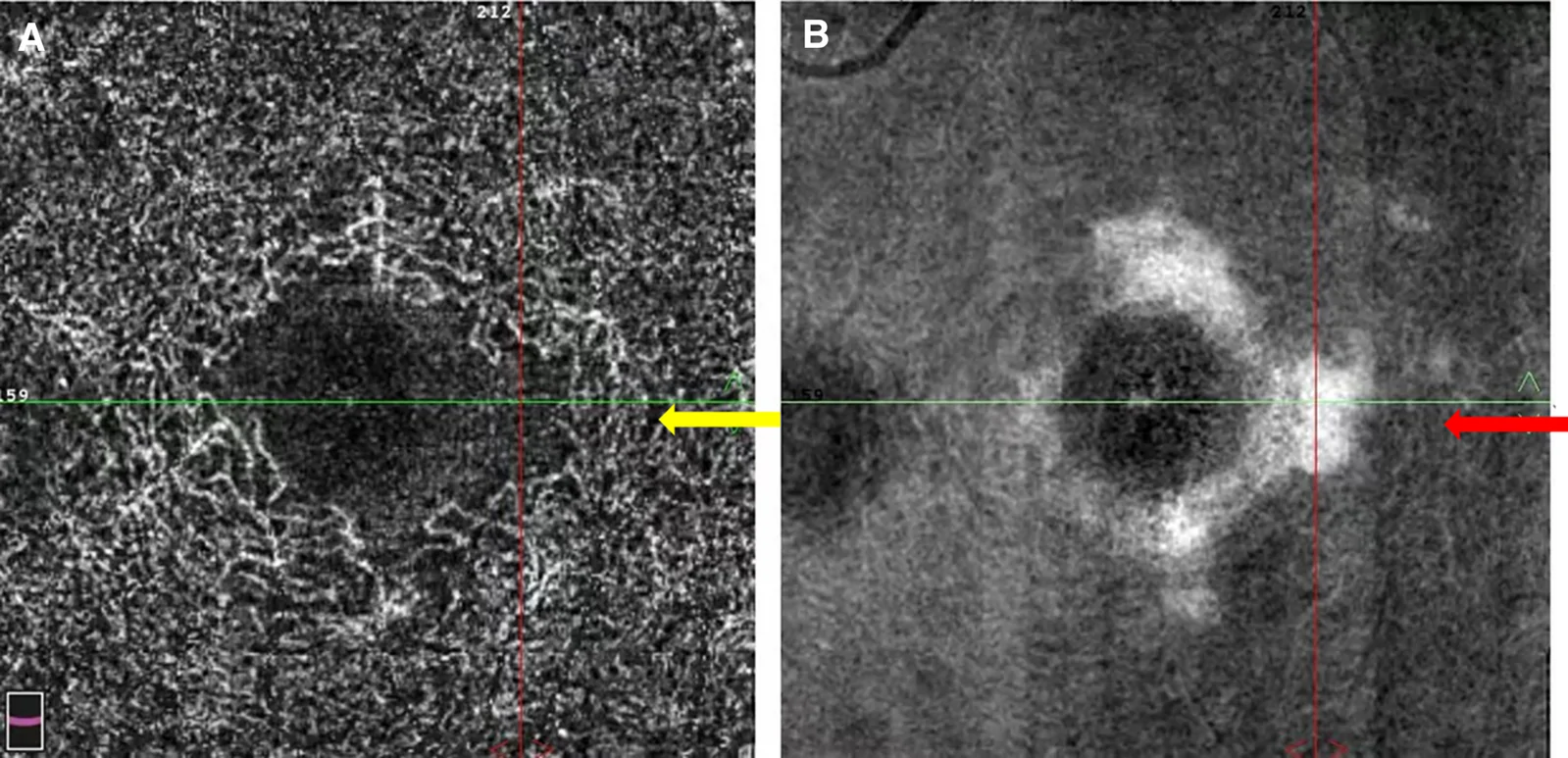
The OCTA examination results of case 1, which reveals a decrease in blood flow density within the deep capillary layer of the retinal fovea on the temporal side (depicted by the yellow arrow). The enface OCT image demonstrates high reflectivity at the corresponding location (marked by the red arrow).

### 3.3. Follow-up (changes in fundus multimodal imaging)

During the follow-up, we found that the range of weak reflective lesions in both eyes gradually decreased in near-infrared fundus photography, and the boundary gradually became blurred. OCT showed that the disruption of the ellipsoid and interdigitate zones (EZ and IZ, respectively) gradually recovered. The changes of OCT in other cases were shown in Figure 5. After 3 months of follow-up in case 2 (Fig. 5A and 5B) and 7 months of follow-up in case 4 (Fig. 5E–5G), there was no obvious abnormality in the OCT structure of both eyes. In case 4, the patient still complained of a dark spot in front of his eyes, so he underwent a multifocal ERG (mfERG) examination, which showed that the amplitude of the macular region in both eyes was lower than normal (Fig. 6).

**Figure 5.**
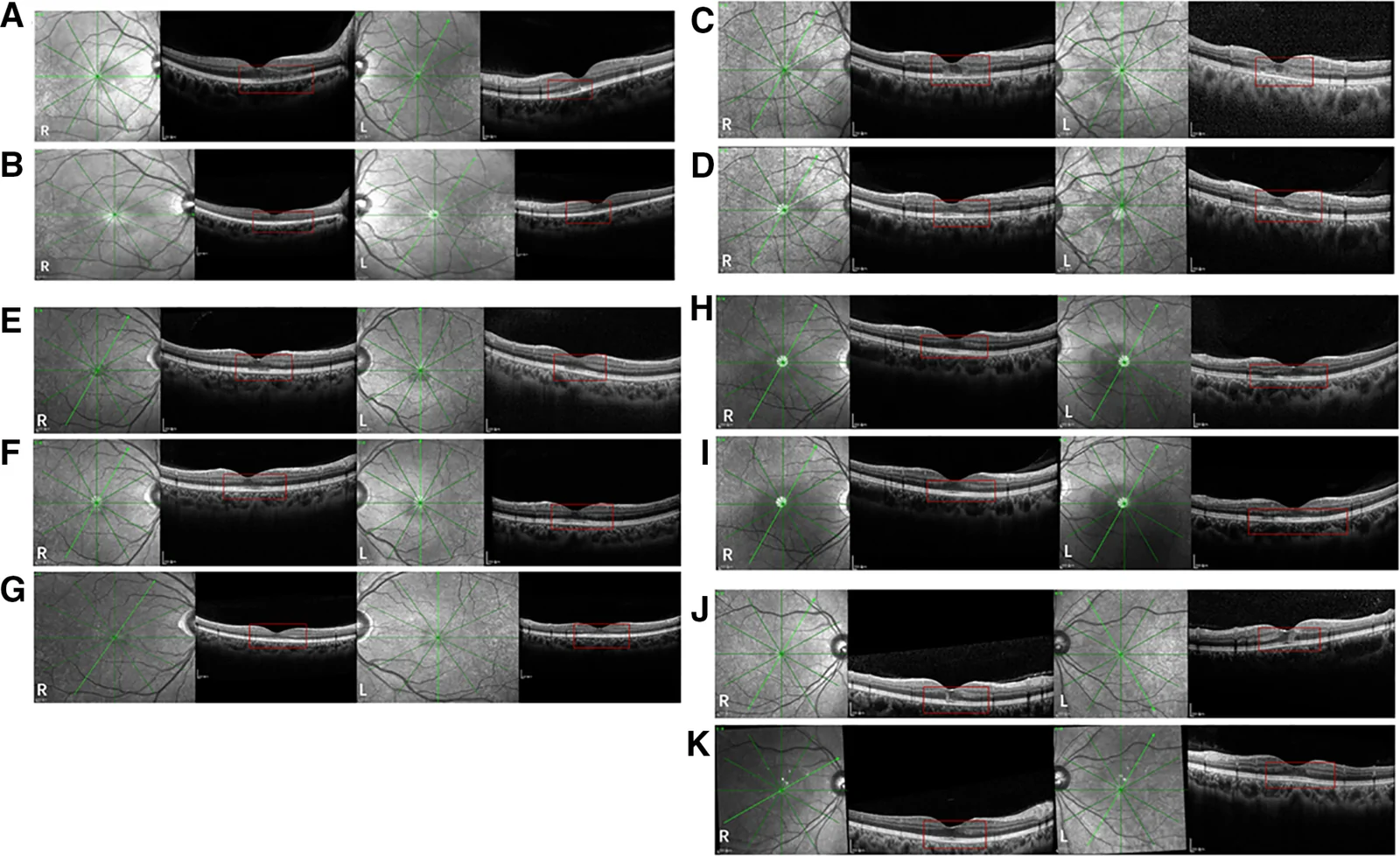
The changes of OCT in cases 2 to 6. (A) OCT images of case 2 at diagnosis. (B) OCT images of case 2 after 3 months follow-up. (C) OCT images of case 3 at diagnosis. (D) OCT images of case 3 after 3 months follow-up. (E) OCT images of case 4 at diagnosis. (F) OCT images of case 4 after 3 months follow-up. (G) OCT images of case 4 after 7 months follow-up. (H) OCT images of case 5 at diagnosis. (I) OCT images of case 5 after 3 months follow-up. (J) OCT images of case 6 at diagnosis. (K) OCT images of case 6 after one month follow-up. L = left eye, R = right eye.

**Figure 6.**
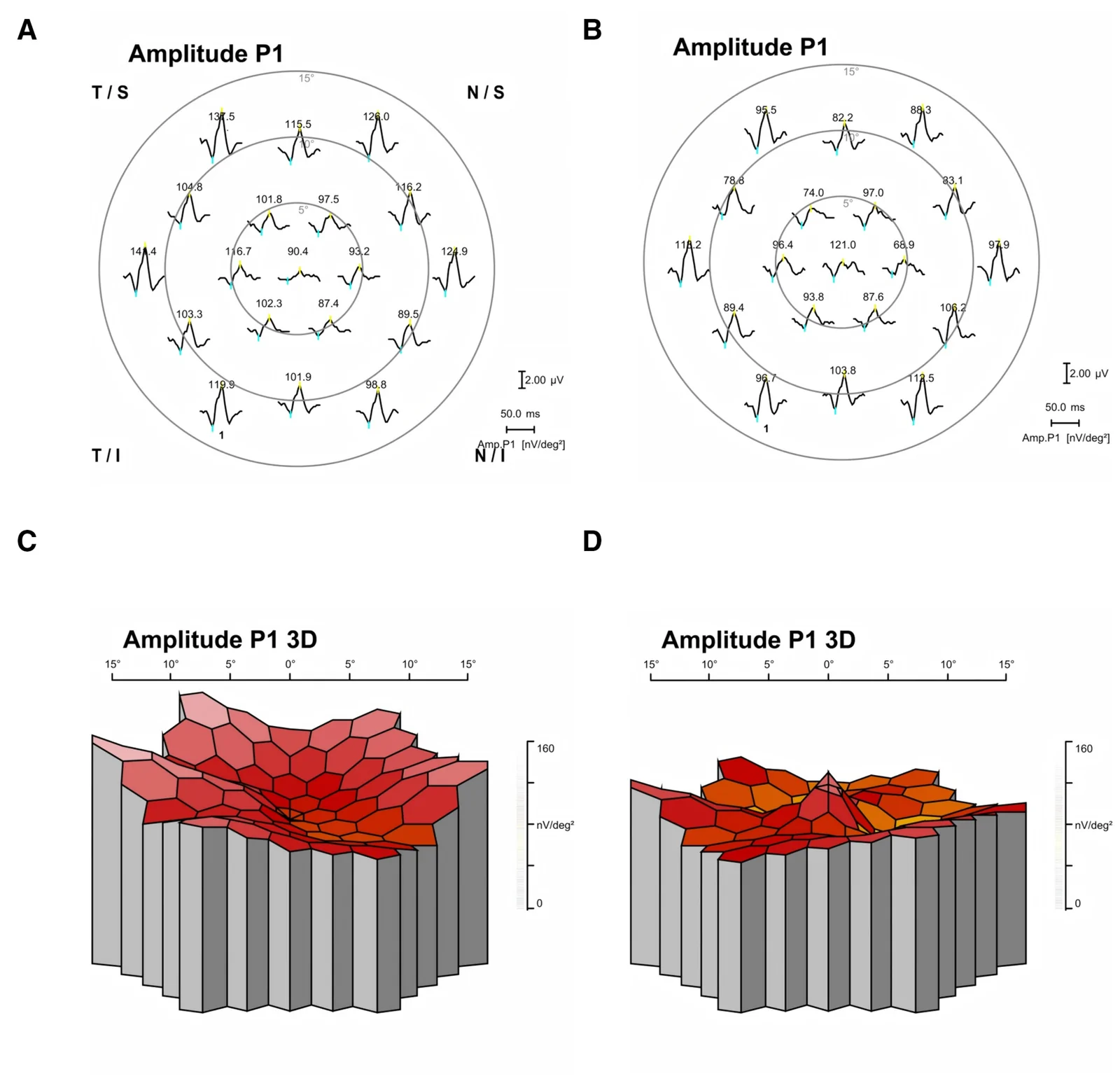
The multifocal electroretinography (mfERG) of case 4. The amplitude in the macular region is lower than the normal value in both eyes. (A) Annular chart of the amplitude in the right eye. (B) Annular chart of the amplitude in the left eye. (C) Three-dimensional diagram of the amplitude in the right eye. (D) Three-dimensional diagram of the amplitude in the left eye.

## 4. Discussion

AMN is a rare retinal macular disease typically characterized by sudden, peripheral, dark spots in one or both eyes, with or without vision loss, mainly affecting young and healthy women.^[5]^ Since its first report in 1975, the pathogenesis of AMN remains unclear. Previous literature has reported that most cases are attributed to initial influenza-like illnesses, but no specific evidence has been established to date.^[6]^ During the SARS-CoV-2 pandemic, SARS-CoV-2 infections are often accompanied by various ocular manifestations, with the most common ocular complication being conjunctivitis, with a prevalence rate of up to 33.3%.^[7]^ There are also increasing reports of retinal vascular and neurological complications associated with it,^[8–10]^ including retinal vein occlusion, retinal vasculitis, optic neuritis, and AMN. Here, we report a case series of typical AMN in young and healthy patients, a sudden increase during the first surge of the SARS-CoV-2 infection immediately after the easing of epidemic control measures in early December 2022 in Zibo, China. In about 2 weeks, as many as 6 cases of AMN presented to our hospital’s outpatient clinic, with a clear trend of increase. Before COVID-19 pandemic, there were only about 0–2 new cases per year in the past decade. A previous study indicated that from December 2022 to February 2023, a total of 78 new cases of AMN were reported based on data from 6 hospitals. By contrast, between December 2021 and February 2022, these institutions had a total of only 2 new cases of AMN.^[11]^ Consistently, another study pointed out that since the COVID-19 outbreak, the incidence rate of AMN in a French hospital has increased from 0.66/100000 in 2019 to 8.97/100000 in 2020 (*P* = .001).^[11]^ The obvious increase in AMN cases after the COVID-19 pandemic, and the fact that all 6 cases were diagnosed after SARS-CoV-2 infection, suggest a possible temporal association between SARS-CoV-2 infection and the occurrence of AMN. This study only demonstrates a temporal association and does not establish causality.

In 17 individual case reports of AMN associated with SARS-CoV-2 infection, patients’ ages ranged from 12 to 71 years old (average age of 25.2 years old),^[12]^which is consistent with this study where the age of onset is in middle and young adulthood. The main clinical symptoms include one or multiple paracentral scotomas in one or both eyes, with or without visual impairment. The typical fundus finding is a well-defined, wedge-shaped dark lesion in the macular area, with the tip pointing towards the fovea. However, many cases have no significant fundus changes, only irregular reflection on the surface of the retina in the macular area, making it difficult to detect abnormalities on FFA examination, which can lead to misdiagnosis or missed diagnosis in many cases. Clinically, typical near-infrared fundus photography and OCT examination can be used for accurate diagnosis. Near-infrared fundus photography can clearly show the appearance of the lesion, typically with low-reflection lesions. OCT examination results show that there are spotty high-reflection lesions in the outer nuclear layer and outer plexiform layer, with local ellipsoid zone defects and discontinuities. This suggests that the lesion is located in the photoreceptor cell layer of the neural retina and may further affect the inner retinal layer as the disease progresses. OCTA can clearly show the retinal blood vessel layers, and some AMN patients can be observed with reduced blood flow density in the deep retinal capillaries corresponding to the lesion site.

The exact mechanism of AMN caused by the virus is still not fully understood, with different hypotheses of pathogenesis, including coagulation abnormalities, excessive inflammation, and immune dysfunction. The main receptor of the SARS-CoV-2 virus is angiotensin-converting enzyme 2 (ACE2). Immunohistochemical studies on human eyes have reported high expression of ACE2 receptors in the ciliary body, choroid, retina, and retinal pigment epithelium.^[13]^ In fact, SARS-CoV-2 RNA has been detected in the retinas of some patients who died from SARS-CoV-2 infection,^[14]^ indicating that retinal cells are inevitably involved. Studies have shown that ACE2 mediates SARS-CoV-2 infection in human cells, which can promote inflammation and lead to a hypercoagulable state, resulting in coagulation disorders and increased degradation of fibrinogen, thus leading to distal ischemia,^[15]^ and subsequently causing various systemic complications, including respiratory failure, myocardial infarction, deep vein thrombosis, and stroke with high mortality rates.^[16]^ It is known that AMN is closely related to deep retinal capillary ischemia,^[17]^ which further suggests a possible link between SARS-CoV-2 infection and AMN. In a comparative experiment of COVID-19-related OCTA examination, compared with the control group, retinal vessel density was significantly altered in COVID-19-infected subjects, with significant reduction in all retinal vessel densities in the macular area.^[18]^ This is consistent with our observation that retinal deep capillary blood flow density was reduced in 2 eyes in OCTA examination. We speculate that endothelial damage and abnormal high expression of ACE2 can lead to hypercoagulation and subsequent thrombotic microvascular disease, which may contribute to deep retinal capillary ischemia and thereby trigger AMN. OCTA can detect microvascular changes in the retina of patients after SARS-CoV-2 infection, which can help detect retinal microvascular damage in asymptomatic patients. Whether this noninvasive imaging technique can serve as an effective marker of systemic vascular dysfunction and indirectly reflect systemic organ dysfunction requires more case studies and multicenter studies for verification.

Several potential confounding factors should be considered when interpreting the findings of this study. These include prior COVID-19 vaccination status, systemic inflammatory response (e.g., high fever), use of medications (such as antipyretics or vasoconstrictive agents), hormonal factors, and other underlying systemic or vascular conditions. Due to the retrospective nature of this study and the limited sample size, these variables were not systematically controlled or analyzed.

A 2016 study of 156 eyes with AMN found that 54% of cases were bilateral in nature.^[6]^ In contrast, patients with SARS-CoV-2 infection and AMN during the pandemic had a higher bilateral prevalence of AMN, with rates of 67% (10/15) in a study by Dinh et al^[19]^ and 100% (14/14) in a study by Liu et al.^[2]^ Our study also found that all cases developed bilateral involvement, consistent with the above-mentioned findings, suggesting that infection with SARS-CoV-2 may predispose individuals to a higher risk of developing AMN, with a higher likelihood of bilateral involvement. Based on these reports and our findings, patients with SARS-CoV-2 infection may have a higher risk of AMN. Ophthalmologists should be aware of the potential for the development of AMN following SARS-CoV-2 infection, despite its rarity, and pay attention to the multimodal imaging features of AMN in the fundus. OCT and NIR are the most sensitive imaging modalities for the diagnosis and follow-up of AMN. OCTA can detect flow voids in the choriocapillaris or deep retinal capillary plexus, which correspond to the wedge-shaped lesions on near-infrared imaging; these findings provide evidence for the ischemic mechanism involved. However, OCTA changes are not always detected. mfERG can reflect the functional status of the affected area of the retina. We observed that although OCT structural abnormalities had resolved, there was still a reduction in mfERG amplitude, and patients reported symptoms of visual field defects, suggesting that retinal function recovery in patients with AMN may be a prolonged process. Even if OCT structural abnormalities normalize, there may still be retinal dysfunction. Retinal function recovery lags behind OCT structural recovery. Therefore, follow-up should not be limited to normal OCT structural findings; it should continue for a longer period to assess retinal function. Potential confounding factors were not controlled, which may influence the observed association.The small sample size limits the generalizability of our findings and precludes robust statistical analysis. Larger, multicenter studies are required to validate these observations.

## 5. Conclusions

Based on our cases, the fundus features of AMN following SARS-CoV-2 infection were as follows: near-infrared fundus photography reveals low-reflection lesions, while OCT shows spotty high-reflection lesions in the outer nuclear and plexiform layers, with ellipsoid zone defects. OCTA indicates reduced blood flow density in the deep retinal capillaries, with enface OCT showing high reflection at the corresponding site. No difference was observed in imaging between AMN after SARS-CoV-2 infection and AMN not related to SARS-CoV-2. However, the increasing cases after COVID-19 infection imply that patients with SARS-CoV-2 infection are at greater risk of developing AMN. Further studies are needed to confirm the association between AMN and SARS-CoV-2 infection and to understand the exact mechanism of the disease.

## Acknowledgments

The authors would like to express their gratitude to all the participants and staff who were involved in this study.

## Author contributions

**Conceptualization:** Chao Sun, Huaihua Wang, Na Liu, Hui Lu, Shengnan Zhang.

**Data curation:** Chao Sun, Huaihua Wang, Na Liu, Hui Lu, Shengnan Zhang.

**Formal analysis:** Chao Sun, Huaihua Wang, Na Liu, Hui Lu, Shengnan Zhang.

**Writing—original draft:** Chao Sun, Shengnan Zhang.

**Writing—review & editing:** Chao Sun, Shengnan Zhang.

**Funding acquisition:** Shengnan Zhang.

**Investigation:** Shengnan Zhang.
